# Value of AFP and PIVKA-II in diagnosis of HBV-related hepatocellular carcinoma and prediction of vascular invasion and tumor differentiation

**DOI:** 10.1186/s13027-020-00337-0

**Published:** 2020-11-23

**Authors:** Yuan-Quan Si, Xiu-Qin Wang, Gang Fan, Chang-Yin Wang, Yuan-Wen Zheng, Xie Song, Cui-Cui Pan, Fu-Lu Chu, Zhan-Feng Liu, Bing-Ru Lu, Zhi-Ming Lu

**Affiliations:** 1 Department of Clinical Laboratory, Shandong Provincial Hospital, Cheeloo College of Medicine, Shandong University, Jinan, 250021 Shandong PR China; 2 Department of Clinical Laboratory, Shandong Provincial Hospital Affiliated to Shandong First Medical University, Jinan, 250021 Shandong PR China; 3 Department of Hepatobiliary Surgery, Shandong Provincial Hospital Affiliated to Shandong First Medical University, Jinan, 250021 Shandong PR China

**Keywords:** HBV-related hepatocellular carcinoma, Alpha-fetoprotein, PIVKA-II, Vascular invasion

## Abstract

**Background:**

To explore the value of alpha fetoprotein (AFP) and protein induced by vitamin K absence or antagonist-II (PIVKA-II) in diagnosis of HBV-related hepatocellular carcinoma (HCC) and their relationship with vascular invasion, tumor differentiation and size.

**Methods:**

A total of 433 participants were enrolled in this study including 266 cases with HBV-related HCC, 87 cases with HBV DNA positive benign liver disease and 80 healthy individuals. Then we explored the correlation between AFP, PIVKA-II serum level and several pathological features such as vascular invasion, tumor differentiation and size. The value of these two markers used singly or jointly in diagnosing HBV-related HCC was evaluated by receiver operating characteristic (ROC) curve. The ROC curve was also plotted to identify AFP, PIVKA-II serum cut-off values that would best distinguish HBV-related HCC patients with and without vascular invasion.

**Results:**

The level of AFP and PIVKA-II in HBV-related HCC group was significantly higher (Z was 7.428, 11.243 respectively, all *P* < 0.01). When AFP and PIVKA-II were used as the individual tumor marker, the areas under the ROC curve (AUC) of HBV-related HCC diagnosis were 0.765 (95% CI, 0.713 ~ 0.8170) for AFP, 0.901 (95% CI, 0.868 ~ 0.935) for PIVKA-II, and 0.917 (95% CI, 0.886 ~ 0.948) for AFP and PIVKA-II simultaneously. The serum levels of AFP and PIVKA-II were positively correlated with tumor differentiation and size. High AFP and PIVKA-II expression was significantly associated with the presence of vascular invasion (*P* was 0.007 and 0.014 respectively). The AFP level > 64.4 ng/ml or PIVKA-II level > 957.61mAU/ml was the best critical value to predict the presence of vascular invasion.

**Conclusion:**

Our results validate that AFP and PIVKA-II play a significant role in the diagnosis of HBV-related HCC. The diagnostic value of AFP and PIVKA-II combined detection or single assay of PIVKA-II is higher than that of separate assay of AFP. Moreover, their concentration has important clinical value in judging tumor size, tumor cell differentiation and vascular invasion.

## Background

HCC is currently the fifth most common malignant tumor and the third leading cause of cancer related death worldwide [1]. There are close relationships among chronic viral hepatitis (such as Hepatitis B and Hepatitis C), posthepatitic cirrhosis and HCC [2–4]. For the lack of typical symptoms of HCC in early stage, it is generally not easy to diagnose. More than 80% HCC patients are diagnosed at the middle or late stages. Therefore, it is urgent to identify effective and specific biomarkers that provide early predictive potential for diagnosis of HCC [5].

AFP is a kind of glycoprotein that is often associated with HCC. However, AFP levels also increase in pregnancy and some benign diseases such as severe hepatitis and cirrhosis. AFP is not significantly increased in about 35% ~ 40% of the HCC patients, especially for small HCC [6, 7].

PIVKA-II is an abnormal form of prothrombin, which has been used as a good diagnostic biomarker for HCC [8–10]. There is now considerable evidence that PIVKA-II is an independent prognostic factor after liver surgery, such as hepatic resection or liver transplantation [11]. In addition, PIVKA-II is influenced by many non-tumor factors, such as coagulation dysfunction, liver cirrhosis and so on [8].

There were few studies on the correlation between the above two indicators and vascular invasion, tumor differentiation and size. And the conclusions are controversial [12–14]. The aim of the study was to evaluate the diagnostic value of PIVKA-II and AFP in the diagnosis of HBV-related HCC and futher analysis the level of AFP and PIVKA-II in HBV-related HCC patients with different clinicopathologic characteristics such as tumor differentiation and vascular invasion.

## Materials and methods

### Patients

Two hundred sixty-six cases of HBV-related hepatocellular carcinoma which were newly diagnosed in Shangdong Provincial Hospital from January 2017 to December 2018 were recruited as experimental group. All cases were confirmed by pathological examination. Pathological report of HBV-related HCC included tumor diameter and differentiation. Vascular invasion was defined by the presence of tumor cells forming a thrombus in a vascular space lined by endothelial cells. Eighty-seven patients with HBV DNA positive benign liver disease (including 47 cases of HBV-related hepatocirrhosis and 40 cases of Hepatitis B) and 80 healthy individuals were selected during the same period as control. Benign liver diseases were ascertained by image tests or ultrasonography detection. There was no evidence of any cancer or inflammatory disease from blood test results with healthy individuals. Participants with Haemocoagulatory disorders, vitamin K uptake disorders and intake of vitamin K blocking agents were excluded. Informed consents were obtained from all participants.

### Measurement of AFP and PIVKA-II

The contents of AFP and PIVKA-II were detected in the same serum samples respectively. AFP was measured using the method of immunofluorescence on automatic electrophoresis fluorescence immunoassay analyzer (mTAS Wako i30, Japan). The cut-off value was set at 20 ng/ml. Serum levels of PIVKA-II were detected by chemiluminescence enzyme immunoassay on automatic chemiluminescence immunoassay instrument (LUMIPULSE® G1200, Japan). The cut-off value was 40mAU/ml. The above reagents required were all original kit and the standard operation procedure was followed strictly during the test.

### Confirmation of vascular invasion

Firstly, microscopic infiltration of the vessels at the periphery of the HBV-related HCC nodules was searched in all native livers by hematoxylin-eosin (H&E) staining. Then, tumor vessel invasion was assessed by identifying neoplastic emboli within the tumor vessels stained by the mouse monoclonal antibody against CD34 protein. The stained slides were blindly and randomly examined by the same pathologist who had already evaluated H&E sections.

### Statistical analysis

IBM SPSS statistical software (version 22.0, USA) and MedCalc (version 15.2.2.0, Belgium) were used for all statistical analysis. It was found that the concentration levels of AFP and PIVKA-II were skew distribution. Each variable was represented as median with interquartile range (IQR). Multi-numerical variables were compared by Kruskal-Wallis test, and the nonparametric Mann-Whitney test was applied to compare the differences between two numerical variables. Log transformation was used for AFP and PIVKA-II values to analyse the large range of values among the different groups for the markers. The transformed data were compared by box plots. Categorical variables were expressed in percentage and compared with the Pearson Chi-square test. ROC Curves and AUROC were calculated to evaluate the diagnostic value of AFP and PIVKA-II singly or jointly. Then Z tests were applied to compare the difference of AUROC. Two-tailed *P* value less than 0.05 was defined statistically significant.

## Results

### Patient demographics and tumor characteristics

The cut-off value of AFP and PIVKA-II commonly used was 20 ng/ml and 40mAU/ml respectively. Based on this critical value, patient demographics and tumor characteristics were outlined in Table 1. As shown in Table 1, we found that AFP and PIVKA-II had strong correlation with tumor size (measured by diameter) and differentiation as well as vascular invasion.

**Table 1:**
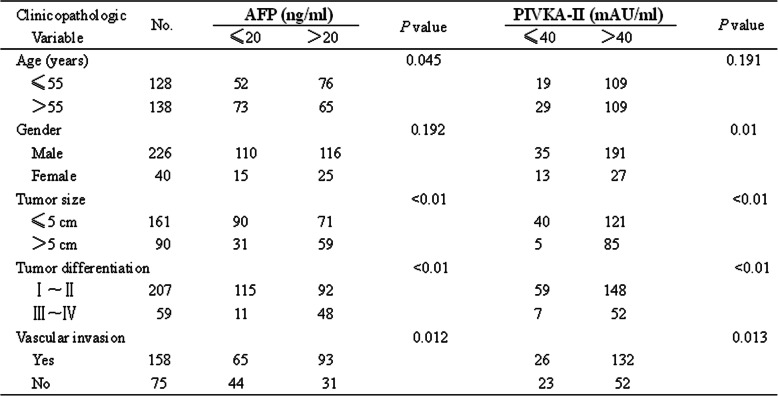
Correlations between AFP and PIVKA-II expression and clinicopathologic variables of HBV-related HCC patients

### AFP and PIVKA-II serum levels in experimental and control group

**Fig. 1:**
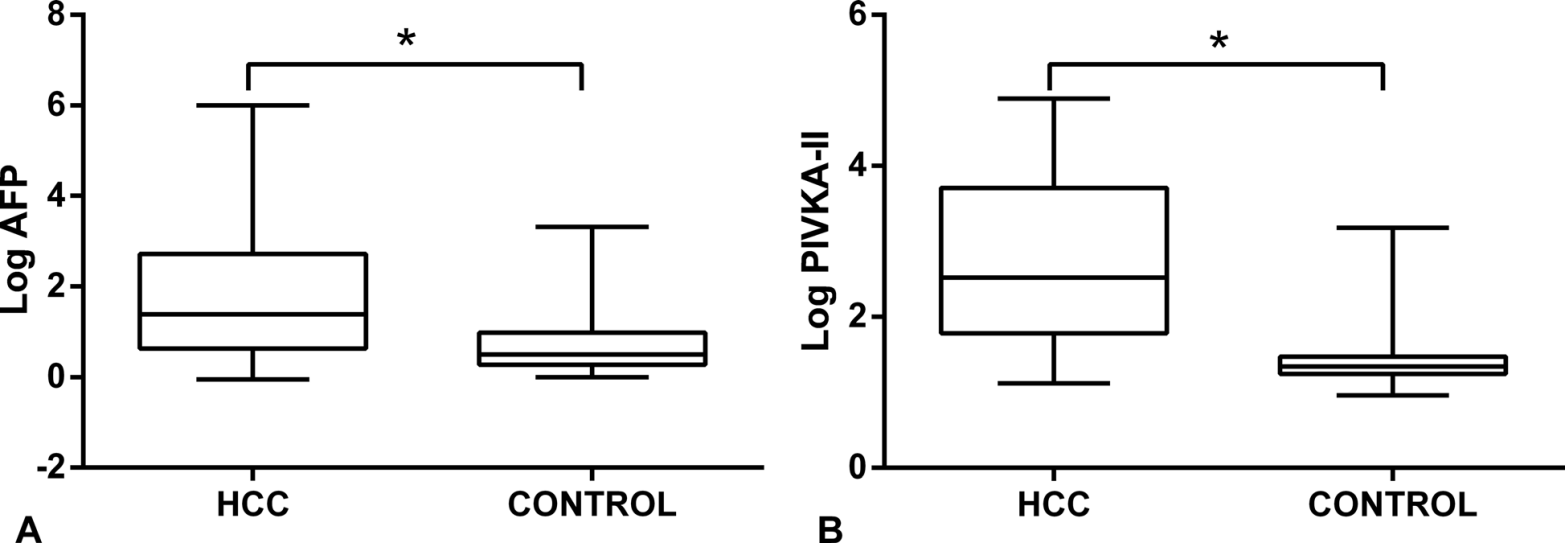
Log transformation was used on the AFP and PIVKA-II values to account for the large range of values for both markers. Log values of serum levels of AFP (**a**) and PIVKA-II (**b**) in HBV-related HCC and control group were compared by box plots. AFP: alpha fetoprotein, PIVKA-II: prothrombin induced by vitamin K absence- II. *P < 0.01

The median levels of AFP and PIVKA-II in HBV-related HCC patients were 24.64 (IQR 4.38 ~ 528.82) and 334.08 (IQR 60.88 ~ 5095.10), while their concentrations of control group were 3.20 (IQR 1.90 ~ 9.60) and 22.17 (IQR 17.59 ~ 30.05) respectively. As shown in the Fig. 1, the AFP and PIVKA-II levels were significantly higher in the HBV-related HCC group than that in the control group (Z was 7.428, 11.243 respectively, *P* all < 0.01). There was no significant difference of serum AFP and PIVKA-II level between patients with benign liver disease and healthy people in the control group. Above results validated that AFP and PIVKA-II played a significant role in the diagnosis of HBV-related HCC.

### Comparison of AFP and PIVKA-II single and combined detection in HBV-related HCC diagnosis

**Fig. 2:**
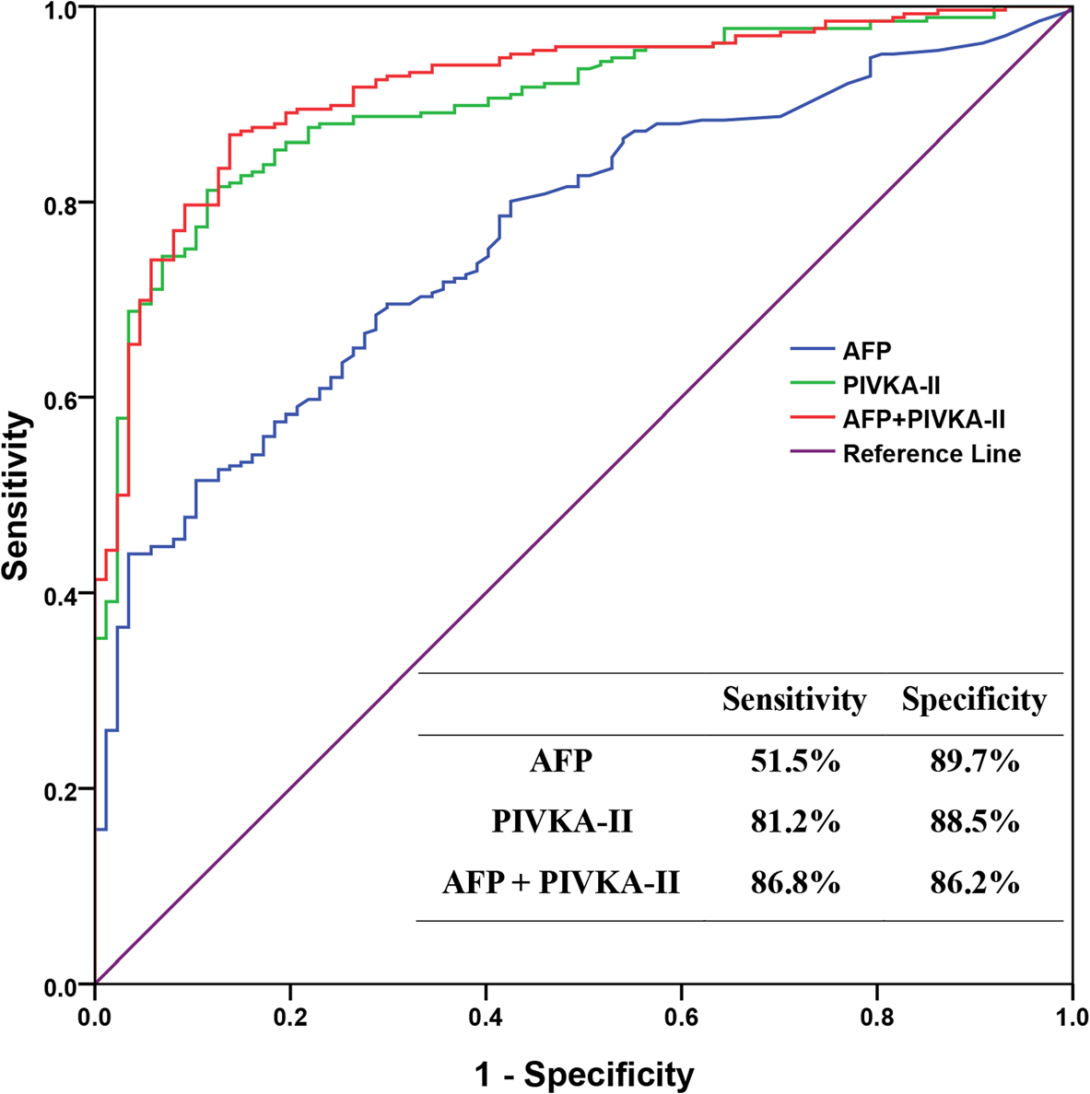
ROCs curve comparing value of AFP, PIVKA-II and the combination of them as diagnostic markers for HBV-related HCC. Sensibility and specificity of the two kind of markers in detecting HBV-related HCC was also listed. AFP: alpha fetoprotein, PIVKA-II: protein induced by vitamin K absence-II

The ROC curve was plotted to compare diagnostic values of AFP and PIVKA-II detected singly or jointly in HBV-related HCC and identify their cut-off values that would best distinguish patients with HBV-related HCC from control group. As pictured in the Fig. 2, the AUC of HBV-related HCC diagnosis was 0.765 (95% CI, 0.713 ~ 0.8170) for AFP, and 0.901 (95% CI, 0.868 ~ 0.935) for PIVKA-II. The combination of AFP and PIVKA-II improved the diagnostic performance for HBV-related HCC (AUC 0.917; 95% CI, 0.886 ~ 0.948). The diagnostic values of AFP and PIVKA-II combined detection or single assay of PIVKA-II were better than that of separate assay of AFP (Z was 5.927 and 4.51 respectively, *P* all < 0.001). There was no significant difference of diagnostic accuracy between joint test of the two biomarkers and PIVKA-II detected singly (Z was 1.795, *P* = 0.0727). The optimal cut-off value of HBV-related HCC diagnosis was 21.8 ng/ml for AFP and 41.74mAU/ml for PIVKA-II. At the optimal cut-off value of AFP and PIVKA-II, sensitivity and specificity in diagnosis of HCC were reflected in Fig. 2.

### Correlations between AFP, PIVKA-II serum levels and tumor differentiation and size (measured by diameter) of HBV-related HCC

**Fig. 3:**
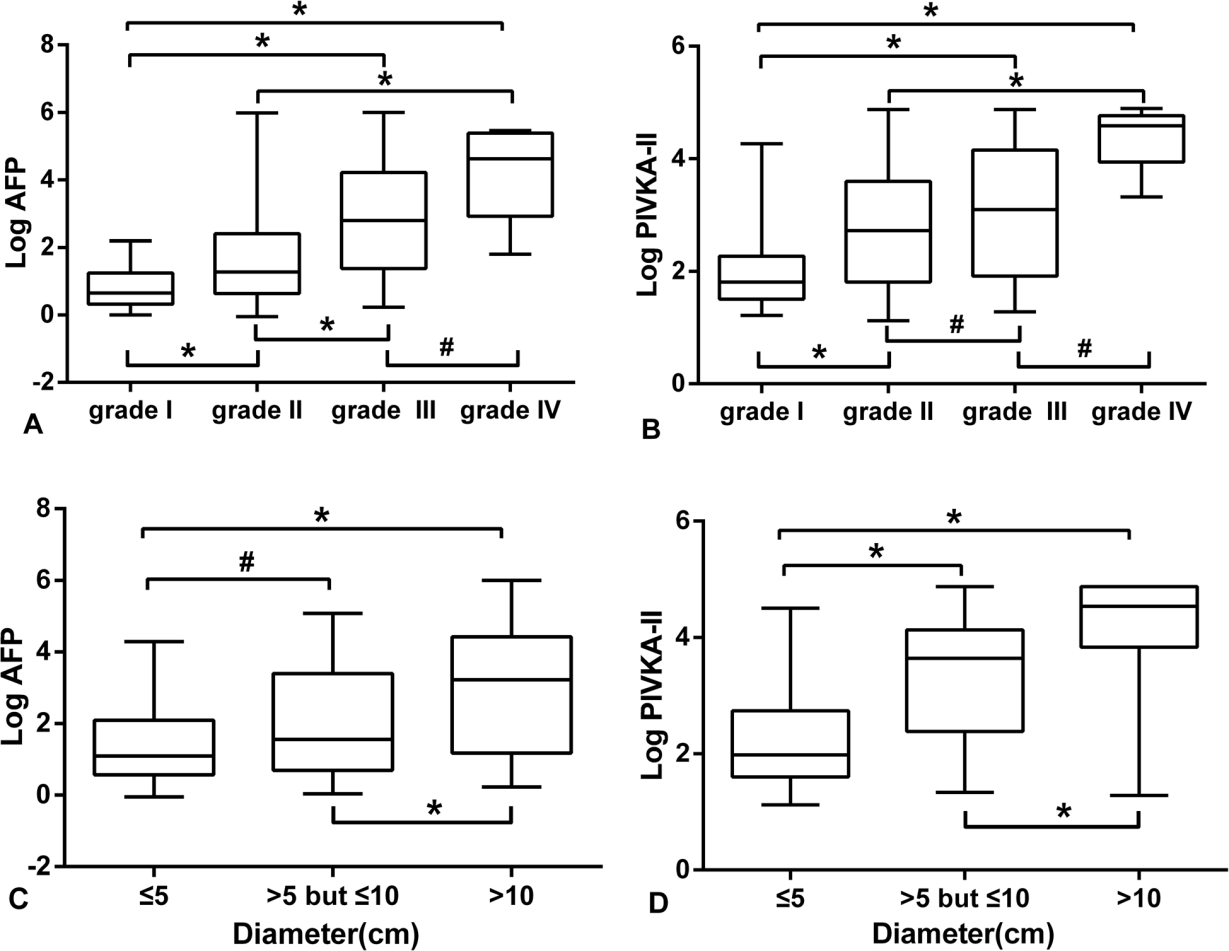
Log values of serum levels of AFP (**a** and **c**) and PIVKA-II (**b** and **d**) were used to compare difference between their serum levels and tumor differentiation and size (measured by diameter) of HBV-related HCC. Among them, grade I and grade II were considered as well and moderate differentiation respectively while grade III or IV were regarded as poor differentiation. Based on the HBV-related HCC diameter, the experimental group were divided into the ≤5 cm, > 5 but ≤10 cm and > 10 cm group. AFP: alpha fetoprotein, PIVKA-II: protein induced by vitamin K absence-II. *P < 0.05 but ^#^*P* > 0.05

The AFP, PIVKA-II concentration showed significant differences in well/moderate/poor differentiation as well as tumor size of HBV-related HCC. Generally, the serum levels of AFP and PIVKA-II were positively correlated with tumor differentiation and size, just as shown in Fig. 3.

### Predictive value of AFP and PIVKA-II for HBV-related HCC vascular invasion

**Fig. 4:**
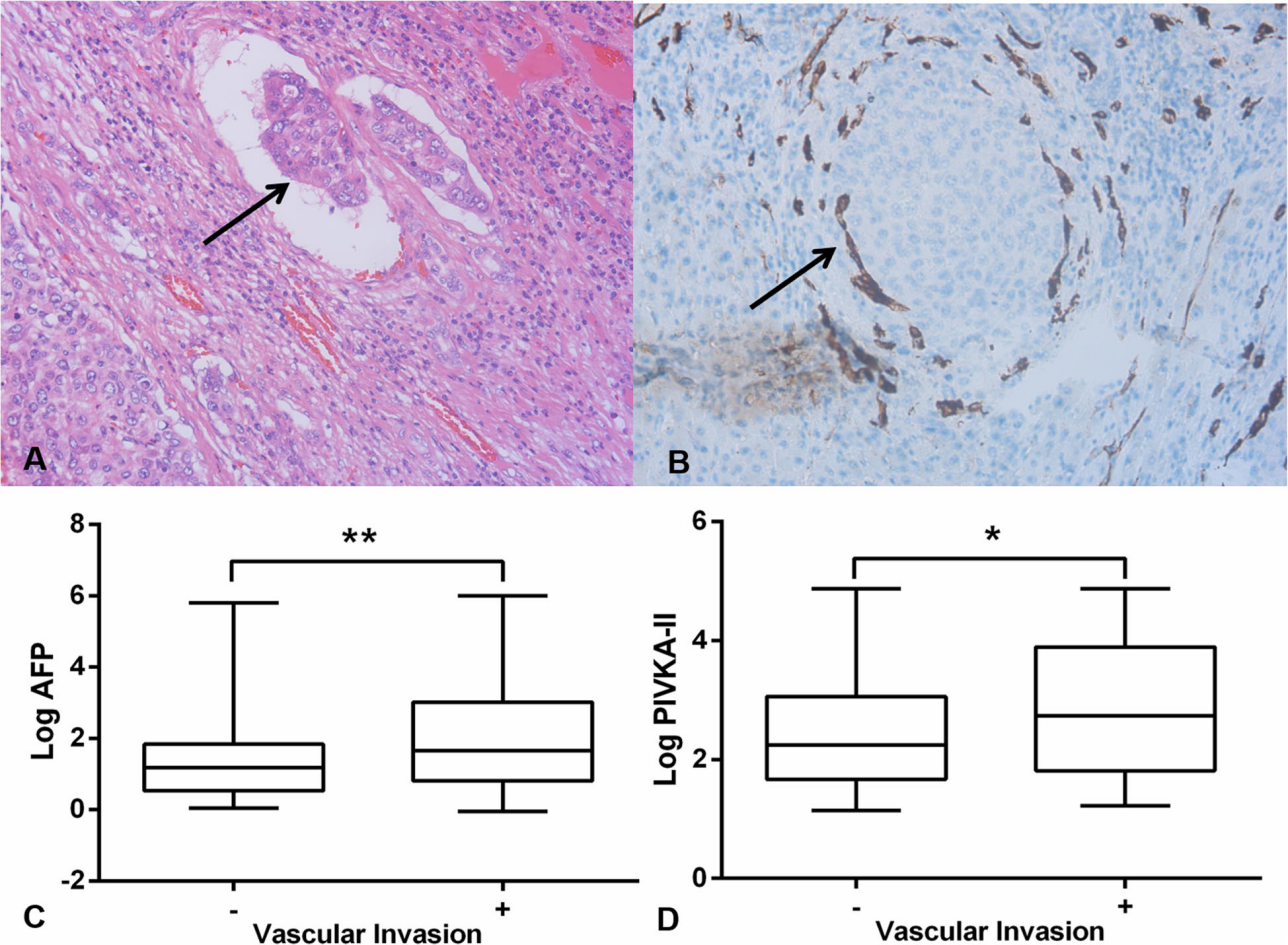
The arrow in (**a**) showed patent microscopic invasion of a vessel by neoplastic cells (H&E staining; magnification X100). With anti-CD34 immunohistochemistry (**b**), vascular invasion was confirmed by the presence of small nests of malignant cells inside the vessels (magnification X200). Log values of serum levels of AFP (**c**) and PIVKA-II (**d**) in HBV-related HCC with and without vascular invasion were compared by box plots. AFP: alpha fetoprotein, PIVKA-II: protein induced by vitamin K absence-II. *P < 0.05 and **P < 0.01

The positive results of H&E and immunohistochemical staining in HBV-related HCC tissues with vascular invasion were shown in Fig. 4 (A and B). The median levels of AFP and PIVKA-II in HBV-related HCC patients without vascular invasion were 15 (IQR 3.4 ~ 69) and 174.71 (IQR 46.76 ~ 1156.6), while their concentrations of patients with vascular invasion were 45 (IQR 6.37 ~ 1039.2) and 549.3 (IQR 65.13 ~ 7805.3) respectively. As shown in the Fig. 4 (C and D), the AFP and PIVKA-II level in HBV-related HCC with or without vascular invasion was obviously different. High AFP and PIVKA-II expression was significantly associated with the presence of vascular invasion (Z was 2.683, 2.463 respectively, *P* = 0.007 and 0.014). The AFP level > 64.4 ng/ml or PIVKA-II level > 957.61mAU/ml was the predictor of vascular invasion.

## Discussion

The incidence of hepatocellular carcinoma is increasing annually, thus early diagnoses and treatments are particularly important. AFP is a glycoprotein that is the most common biomarker for diagnosing HCC. But it has not yet become an optimal biomarker for diagnostic purposes because it lacks high sensitivity and specificity [15]. PIVKA-II also known as des-gamma-carboxyprothrombin (DCP) is an immature form of prothrombin without any coagulative function [16]. PIVKA-II is a specific marker for HCC, which is poorly related to AFP and exhibits higher sensitivity and specificity than AFP in diagnosing HCC [17, 18].

In this study, we evaluated the performance of AFP and PIVKA-II for the diagnosis of HBV-related HCC and explored the relationship between them and different clinicopathologic features, such as vascular invasion, tumor differentiation and size. Compared with control group, the level of AFP and PIVKA-II in experimental group was significantly higher. Our results validated that AFP and PIVKA-II played a significant role in the diagnosis of HBV-related HCC. The optimal cut-off values of HBV-related HCC diagnosis in our study were 21.8 ng/ml for AFP and 41.74mAU/ml for PIVKA-II respectively, which were in close agreement with their cutoff values used currently. The combination of AFP and PIVKA-II slightly improved the diagnostic performance, and the serum PIVKA-II level had a better diagnostic value than AFP. These findings were in accordance with conclusions from other studies [19–23]. The serum levels of AFP and PIVKA-II were positively correlated with tumor differentiation and size. High AFP and PIVKA-II expression was significantly associated with the presence of vascular invasion. The AFP level > 64.4 ng/ml or PIVKA-II level > 957.61mAU/ml was the best critical value to predict the presence and absence of vascular invasion.

The conclusions about the relation between AFP, PIVKA-II and clinicopathologic features (such as vascular invasion, tumor differentiation and size) were different, even controversial [12–14, 24–27]. There were two main reasons for the different conclusions of previous studies. One important factor was research cancer object which was not strictly screened. Hence they did not distinguish the amount and source of tumors. The other reason was related to detection instruments and methods, the levels of AFP and PIVKA-II varied according to instruments and methods used. Based on the above two reasons caused the contradiction between different research. Tumor subjects in our study were HBV-related primary liver cancer, then immunofluorescence assay and chemiluminescence immunoassay were used to measure AFP and PIVKA-II respectively. The methods were accurate which had the advantages of good repeatability and high test efficiency. At the same time, when studying the relationship between AFP, PIVKA-II level and tumor size, all the selected HCC cases were all single tumors and multiple tumors were excluded by imaging examinations. Accordingly, the conclusions were more persuasive. However, data of this study were obtained in a single hospital, and the number of participants was small. Further research should spend more time and get more detailed information, for instance, we were not sure whether PIVKA-II correlates with size or grade better than AFP.

## Conclusions

In conclusion, PIVKA-II is the most useful single biomarker to diagnose the presence of HBV-related HCC. Combining the AFP and PIVKA-II further improves the diagnostic performance. Moreover, their concentrations play an important role in judging tumor size, tumor cell differentiation and vascular invasion.

## Data Availability

The datasets used and/or analysed during the current study are available from the corresponding author on reasonable request.
